# HRMAS NMR Spectroscopy to Identify the Primary Metabolome of Bracigliano PGI Sweet Cherries and Correlate It with Nutraceutical and Quality Parameters

**DOI:** 10.3390/foods14122120

**Published:** 2025-06-17

**Authors:** Domenico Liguori, Pierluigi Mazzei

**Affiliations:** Department of Pharmacy, University of Salerno, 84084 Fisciano, Italy

**Keywords:** metabolomics, nutraceutical, molecular fingerprint, multivariate statistical analyses, Protected Geographical Indication—PGI, semi-solid state NMR

## Abstract

In 2023, the Italian Bracigliano sweet cherries were awarded the important European label PGI. However, reliable information on the compositional and nutraceutical quality of this product is still relatively undefined and fragmented. Therefore, we investigated fresh Bracigliano PGI cherries (Pallaccia, Spernocchia, and Principe varieties) via HRMAS NMR spectroscopy in the semi-solid state, even though it represents an innovative and powerful technique that is still drastically unexplored. We demonstrated the HRMAS NMR suitability for this fruit type as well as identified the primary metabolome of studied Bracigliano PGI types. Moreover, chemometric techniques (ANOVA, PCA, and PLS-DA) permitted the significant definition of a variety-specific compositional fingerprint. HRMAS data were associated with the assessment of chemical and nutraceutical quality parameters. Importantly, in all studied varieties, a relatively high content of total phenols and antioxidant agents was detected, with Pallaccia cherries resulting as the healthiest ones. The heatmap clusterization revealed interesting correlations between HRMAS-NMR data and important quality parameters. Our results confirm the role of HRMAS in food chemistry and invite the creation of a spectral database of Bracigliano sweet cherries, useful to conduct traceability studies, protect consumers from frauds, and bolster the producers in promoting and certifying the quality of their products.

## 1. Introduction

The sweet cherry is the fruit of *Prunus avium* L., an arboreal species belonging to the *Rosaceae* family [1]. It is a very popular food product all over the world due to its pleasant flavor, which can range from very sweet to slightly sour, depending on both variety and ripening extent. In the last 16 years, the production and annual consumption of cherries in the world has increased from 1.9 to 2.32 million tons [2]. The sweet cherry is widely used, even at an industrial level, as both fresh and processed (juices, fruit in syrup, brandy, liqueurs, and preserves) food [3]. The fruit exhibits a juicy pulp and a color ranging from red to a very dark burgundy. The consumption of sweet cherries is reported to bring health benefits mostly because of their capability to contrast the onset of diseases, such as cardiovascular disease, Alzheimer’s disease, inflammatory diseases, and chronic disorders characterized by increased oxidative stress (i.e., cancer and diabetes) [4].

Considering that, after Turkey, the United States, and Iran, Italy represents the fourth nation for cherry production, the Italian producers are paying an ever-increasing attention to high-quality sweet cherries. In particular, in 2023, several varieties of sweet cherries produced in Bracigliano (Salerno, Southern Italy) have been awarded the prestigious PGI (Protected Geographical Indication) due to their peculiar and valuable organoleptic and productive characteristics. This notwithstanding, reliable information on the compositional and nutraceutical quality of this product is still poorly defined and requires a more in-depth characterization. In light of the recent PGI label assignment, it would be amenable to evaluate and report the most relevant traits of the Bracigliano sweet cherries, identifying their main chemical, nutraceutical, and compositional characteristics.

In particular, the HRMAS (High Resolution Magic Angle Spinning) Nuclear Magnetic Resonance (NMR) spectroscopy is an alternative and innovative NMR technique that offers multiple advantages [5,6]. In particular, it allows the analysis of solid, fresh, and hydrated samples without the need to resort to any pre-treatment or expensive and time-consuming preliminary extractions, providing, at the same time, qualitative and quantitative information on the molecular composition of fruits and agricultural products [7]. In spite of the enormous potential of this powerful and reliable analytical technique, it is still very underutilized in agricultural and food chemistry. This is due to several reasons, such as that many researchers are not fully aware of the HRMAS potential; it implies supplementary costs to purchase the probe and related hardware components, and it requires a specific personnel formation. However, the HRMAS advantages are unprecedented and indisputable, especially when the nature of investigated materials makes them susceptible to deterioration during the extraction phases. It is further noteworthy that any study reports the use of HRMAS NMR spectroscopy for the analysis of sweet cherries. In fact, the scarce body of literature concerning the application of NMR spectroscopy to investigate sweet cherries is entirely based on the traditional liquid-state NMR spectroscopy of berry extracts. For example, liquid-state ^1^H-NMR spectroscopy has been combined with multivariate statistical analyses to correlate the metabolomic composition of selected Italian sweet cherries (from the Emilia-Romagna and Apulia regions) with their geographical origin [8]. Analogously, the postharvest changes in the cherry peel metabolome and phytochemical profile have also been assessed [9]. Specifically, Goulas and coworkers have monitored the nutraceutical quality and the NMR-based compositional profile of two Greek cherry cultivars at the harvest and several days after the harvest [10]. Girelli and coworkers have compared the metabolic profiles of two fruit juices obtained from two Apulian cherry cultivars by ^1^H-NMR spectroscopy [11]. NMR spectroscopy has also been used to determine the quality of sour cherry juices from multiple Danish cultivars [12].

Therefore, the principal objective of this study was to verify, for the first time to the best of our knowledge, the suitability of the HRMAS NMR technique to analyze fresh berries of sweet cherries in the semi-solid state. In particular, we focused on some of the most representative varieties of Bracigliano PGI sweet cherries, namely, Spernocchia, Pallaccia, and Principe, which were all produced in 2021 under identical pedoclimatic conditions. The specific targets were (1) to identify the HRMAS-based primary metabolome of Bracigliano PGI sweet cherries; (2) to identify significant and peculiar differences among the investigated cherry varieties through a metabolomic approach; and (3) characterize the most relevant chemical, commercial, and nutraceutical properties of Bracigliano PGI sweet cherries and correlate them with HRMAS results.

## 2. Materials and Methods

### 2.1. Cherry Samples and Analysis of Soluble Solids and Fruit Hardness

The investigated sweet cherries belonged to the varieties Spernocchia (SPE), Pallaccia (PAL), and Principe (PRI) (more information in Supporting Table S1). The berries were collected in orchards located in the city of Casale di Bracigliano (Salerno, Italy; latitude of 40°49′33″ and longitude of 14°41′44″), included within the PGI Bracigliano cherry areal, and situated at an altitude of 410 m above sea level. The orchards were situated on relatively deep soils, characterized by a satisfying level of organic matter as well as good physical and hydraulic properties. All the cherry trees were grafted onto a local rootstock that had been propagated sexually via seed. The harvesting period, which varied according to the variety and was recognized based on the fruit color tone, ranged from 15 May 2021 to 10 June 2021. For each variety, the berries were collected from at least five trees, which were randomly distributed on orchard soil and merged casually to fill several 0.5 kg buckets. Part of the berries was used to evaluate, on-site and at the harvest day, the soluble solids content (°Bx, via digital refractometer HANNA HI196801) and the epicarp hardness (g/cm^2^, via mechanical penetrometer TURONI 53,207 equipped with a 6 mm pin) (25 replicates per variety). The remaining berries (around 1.5 kg) were immediately frozen in liquid nitrogen and then stored at −80 °C until the subsequent chemical and nutraceutical analyses.

### 2.2. Dry Weight pH and Titratable Acidity

The dry weight was measured by putting, for each variety, ten sweet cherries into two separate crucibles (two per variety), which were then placed in an oven at a temperature of 70 °C (TCN 200 PLUS Oven with natural convection; Levanchimica, Bari, Italy) for at least 48 h, until the attainment of a stable weight. The pH and the titratable acidity were measured on cherry juices obtained by pressing fifteen cherries per variety (three extraction replicates per variety) and filtered through a cellulose acetate filter (Whatman 42, pore size 2.5 µm, thickness 200 µm) under vacuum (Laboport KNF, 840.3; Levanchimica, Bari, Italy). In detail, the titratable acidity (expressed as g/L of malic acid equivalents) was determined by titrating the cherry juices (diluted 1:50 *v*:*v* with ultrapure water) with a 0.1 M NaOH solution in the presence of the indicator phenolphthalein, while the pH was examined by potentiometric method (HANNA INSTRUMENT HI5221; Levanchimica, Bari, Italy).

### 2.3. Extraction and Assessment of Nutraceutical Agents

Aiming to extract the nutraceutical agents, six cherries per variety were thawed, pitted, and blended through an immersion mixer. Then, 5 g of cherry puree were put in contact with a hydroalcoholic solution (70% ethanol:distilled water, *v*:*v*) [6]. The suspension underwent a 4 min long ultrasonication (ARGO LAB DU-32; Levanchimica, Bari, Itlay) at a temperature of 30 °C prior to being left to extract for 17.5 h under continuous magnetic agitation and in the absence of light. Subsequently, the suspension was centrifuged (15 min at 6000 rpm) to recover the supernatant, which was filtered through a cellulose acetate filter (Whatman 42, with a pore size of 2.5 µm and a thickness of 200 µm; VWR, Milan, Italy) under vacuum. The extracts were stored at a temperature of −20 °C until subsequent analyses. Three extractions were performed per variety.

The total phenols were measured in the ethanolic extract by using the Folin–Ciocalteu method [13]. The reaction took place according to the progressive addition of 100 μL of cherry extract, 0.5 mL of Folin–Ciocalteu reagent (diluted 1:3 with distilled water), and 0.5 mL of 10% (*w*/*w*) of a sodium carbonate aqueous solution. The mixture was vortexed and kept in the dark for 4 min. Then, the reaction solution was diluted with 4 mL of distilled water, vortexed, and allowed to react in the dark for 2 h and 30 min. Each reaction cycle included the analysis of a blank, conducted by replacing the 100 μL of extract with 100 μL of the hydroalcoholic extractant solution. The total phenol content was quantified by analyzing the reaction mixture at a wavelength of 765 nm with a spectrophotometer (Thermo Spectronic 20 Genesys; Fisher Scientific, Segrate, Milan, Italy). The absorbance data were then compared to a calibration curve obtained by launching the reaction with known and increasing concentrations of gallic acid. The results were finally expressed in mg of gallic acid equivalents (GAEs) per 100 g of fresh weight. Four replicates were performed for each reaction set.

The content of antioxidant agents was assessed in the ethanolic extracts via the 2,2-diphenyl-1-picrylhydrazyl (DPPH) free radical scavenging activity assay [14]. The reaction consisted of the progressive addition of 3 mL of methanol, 0.5 mL of ethanolic extract, and 0.4 mL of a DPPH solution (2.18 mM in methanol). After the addition of each component, the solution was vigorously vortexed for ten seconds and left to react in the absence of light for 30 min. Each reaction set included a blank analysis, in which 0.5 mL of sweet cherry extract was replaced with 0.5 mL of the hydroalcoholic extractant solution. The residual quantity of DPPH was then quantitatively estimated using a spectrophotometric technique, analyzing the samples at a wavelength of 517 nm (Thermo Spectronic 20 Genesys; Deltek, Napoli, Italy). The absorbance data were then compared with a calibration curve built up by conducting the reaction with increasing concentrations of ascorbic acid (r^2^ > 0.99). The results were finally expressed in microgram (μg) of ascorbic acid equivalents (AAEs) per 100 g of fresh weight. Four replicates were performed for each reaction set.

Ethanol (96%, analytical grade), methanol (99.8%, analytical grade), Folin–Ciocalteu reagent, and DPPH reagent were supplied by VWR (Milan, Italy), while gallic acid, ascorbic acid, and sodium carbonate were provided by Merck (Rome, Italy).

### 2.4. HRMAS NMR Spectroscopy

For the HRMAS NMR analyses, cherry samples were removed from the ultra-freezer at −80 °C, allowed to partially thaw at room temperature (for around 20 min), and cut in half. Then, 30 mg of berry mesocarp were softly placed into a 4 mm zirconia HRMAS NMR rotor (50 μL with an internal insert and an external winged cap made of Kelf^®^). A phosphate buffer solution, adjusted to pH 6.8 with potassium phosphate salts (K_2_HPO_4_ and KH_2_PO_4_) and prepared with deuterated water (99.8% D2O/H2O, Armar Chemicals, Paris, France), was poured to suspend the sample into the rotor up to fill all the internal volume [15]. HRMAS NMR spectroscopy was performed at a temperature of 21 °C on a 500 MHz Bruker magnet (Bruker Biospin, Rheinstetten, Germany) equipped with a 4 mm HRMAS probe and operating at resonance frequencies of 500 and 125 MHz for ^1^H and ^13^C nuclei. For each analysis, the rotor was inserted into the magnet and spun at a speed of 5000 ± 5 Hz. Proton NMR spectra were acquired employing the presaturation technique to suppress the aqueous solvent signal and setting 16 k acquisition points, 5 s of recycle delay, 8 dummy scans, and 256 scans. Seven berries per variety were prepared and examined.

For the identification of the most intense NMR signals, 1D ^13^C spectra (inclusive of the proton decoupling technique) and 2D homonuclear correlation spectra such as COSY (correlation spectroscopy), TOCSY (total correlation spectroscopy), and NOESY (nuclear Overhauser effect spectroscopy), as well as ^1^H-^13^C heteronuclear correlation spectra like HSQC (heteronuclear single-quantum coherence) and HMBC (heteronuclear multiple-bond coherence), were acquired on representative samples. All 2D spectra were performed using 16 dummy scans, 64 scans, 2048 acquisition points for 256 experiments, and a spectral window of 16 and 300 ppm for the hydrogen and carbon nuclei, respectively. The mixing times for TOCSY and NOESY were set at 0.05 and 0.8 s, respectively. The heteronuclear spectra were optimized considering a scalar and long-range coupling of 145 and 8 Hz, respectively. Each spectrum was processed using Bruker Topspin 4.1 software. Specifically, the 1D ^1^H spectra were subjected to a line broadening of 0.3 Hz and zero filling with a quadruple factor. Subsequently, phase and baseline corrections were applied to each spectrum. A periodic probe calibration with the KBr was conducted to ensure effective magic angle spinning at 54.7°.

### 2.5. Multivariate Analysis

The most intense signals detected in the ^1^H NMR spectra of examined cherry berries were assigned to individual molecules. Then, the proton spectral range included within 7.766 and 0.764 ppm was divided into 47 buckets, delimiting compound-specific singlets or multiplets, and then integrated. The resulting matrix was composed of 47 variables (spectral buckets) and 21 observations (7 replicates for each variety). For each ^1^H NMR spectrum, the area resulting from the integration of each bucket was normalized by dividing it by the integration of the whole spectrum (integrations expressed as percentages).

Principal Component Analysis (PCA) was used to evaluate HRMAS data, aiming to identify variety-specific peculiarities in the primary metabolome and find variables (loading vectors) discriminating the different types of cherries. The one-way ANOVA (analysis of variance) was used to examine and select the most important and significant loading vectors associated with the explored principal components of PCA. In all cases, only loading vectors capable of contributing significantly (at a *p*-value of at least <0.05 for both ANOVA Tukey and Benjamini–Hochberg tests; α confidence level of 0.05) to the discrimination among the sweet cherry varieties were considered [16,17]. Similarly, the ANOVA test was also exploited to evaluate the significance in the differences observed for chemical and nutraceutical data.

The Partial Least Square–Discriminant Analysis (PLS-DA) was applied to assess the ability to objectively and significantly (at an α confidence level of 0.05) discriminate the different cherry varieties by providing the extent (%) of correct sample classification and the receiver operator characteristics (ROCs) curve. In detail, the cross-validation test was carried out by considering only the HRMAS NMR variables identified by ANOVA as significantly involved in the samples’ inter-class differentiation. This choice had the purpose of reducing calculation time and avoiding redundancy problems. The criterion consisted of considering 5 observations for the training set (samples were classified *a priori*) and the remaining 2 for the test set (samples were left unknown) [16,17]. The cross-validation was repeated 5 times by randomly inverting, per each cherry type, the samples included in the training and test sets. Similarly, PLS-DA was further conducted on a second matrix also including the nutraceutical and chemical data.

The latter data matrix was processed via clustered heatmap, which was performed by centering and reducing the values and displaying them according to a color scale, which ranged from dark red (relatively higher/more concentrated) to light blue (relatively lower/less concentrated) through white (intermediate values). Samples were clustered using Euclidean distance and the Ward’s aggregation method [18]. The Pearson correlation matrix was used to assess the relationship among the variables mostly involved in the discrimination between the sweet cherry varieties and was reported by highlighting in red and light blue the positive and negative significant correlations, respectively. All the statistical applications reported in this study were executed through the XLStat software (v. 2016, Addinsoft, Paris, France).

## 3. Results and Discussion

### 3.1. HRMAS-NMR Spectroscopy

#### 3.1.1. Identification of the Primary Metabolome of Bracigliano PGI Cherries

**Figure 1:**
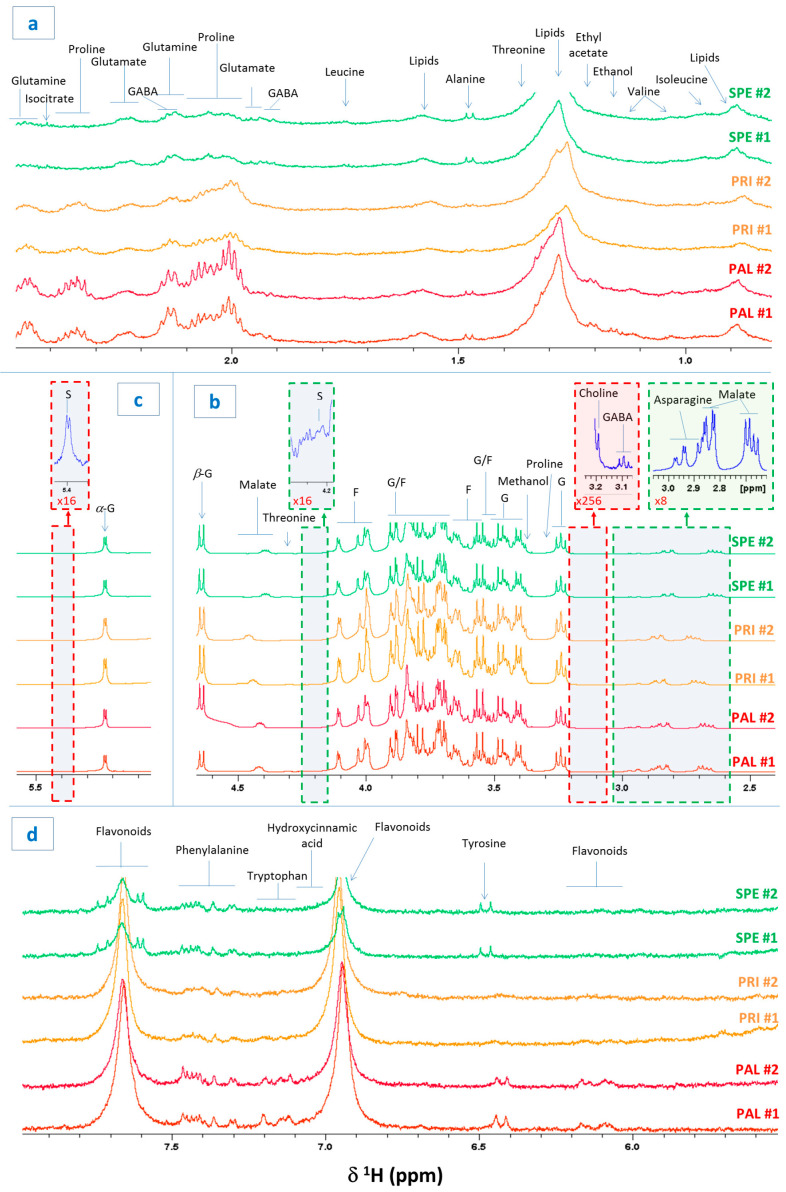
^1^H HRMAS NMR spectra of cherry berries belonging to the Bracigliano PGI varieties Spernocchia (SPE, green), Principe (PRI, yellow), and Pallaccia (PAL, red). The figure shows two replicates per variety (#1 and #2) as well as the spectral regions 0.8–2.5 ppm (**a**), 2.4–4.65 ppm (**b**), 5–5.6 ppm (**c**), and 5.5–8 ppm (**d**). The assignments of the most intense peaks are reported where the labels G, F, S, and GABA stand for glucose, fructose, sucrose, and γ-aminobutyric acid, respectively.

Sweet cherry berries from Spernocchia (SPE), Principe (PRI), and Pallaccia (PAL), PGI Bracigliano plant varieties, were harvested from orchards developed on the same soil, under identical pedoclimatic conditions. The berries were analyzed through HRMAS NMR spectroscopy in the semi-solid state. The latter one represents a very innovative and powerful technique that, despite its indisputable potential, is still underutilized in the fields of food and agricultural chemistry. HRMAS permits obtaining NMR spectra by the direct examination of fresh and intact tissues relatively rapidly and with a resolution very similar to that attainable via NMR in liquid state and high resolution [7]. The ^1^H NMR spectrum of a representative Bracigliano PGI berry is shown in Figure 1. The figure shows two replicates per variety (#1 and #2) and the most relevant spectral regions. In particular, Figure 1a shows the region between 0.8 and 2.5 ppm, which includes proton signals from alkyl groups, either simple or linked to deshielding functional groups. Figure 1b,c display the region between 2.4 and 5.6 ppm, predominantly characterized by hydroxylated alkyl groups (mainly carbohydrates and alcohols) and hydrogens involved in simple double bonds. Figure 1c exhibits the spectral region between 5.5 and 8 ppm, where signals primarily attributable to aromatic groups are found. One of the objectives of this study was to exploit HRMAS NMR spectroscopy to evaluate the primary metabolome of Bracigliano PGI sweet cherries and identify their typical molecular fingerprint. The most intense protonic signals were assigned (Figure 1) based on (1) the previous inherent literature [9,11,12,14] (2) the analysis and interpretation of 2D homo- and hetero-correlated spectra acquired for representative Bracigliano cherry samples and (3) the comparison with NMR spectral databases of certified metabolites. The most abundant amino acids included valine, isoleucine, threonine, alanine, leucine, gamma-aminobutyric acid (GABA), glutamate, glutamine, asparagine, proline, tyrosine, phenylalanine, and tryptophan, whereas the most abundant carbohydrates consisted of glucose, fructose, and sucrose. Additionally, the proton NMR spectra also permitted the identification of alcohols (methanol, ethanol, and choline) and both simple and esterified organic acids (malic acid, typically highly abundant in cherries, and ethyl acetate). For certain signals, a precise assignment was not possible due to excessive broadening, lack of informative multiplicity, or weak signals in 2D experiments. In these cases, when possible, at least the molecular class was reported. Specifically, broad multiplets centered around 0.9 and 1.3 ppm were attributed to methyl and methylene groups of fatty acids, respectively (Figure 1a), while several non-amino acid signals observed in the aromatic region (Figure 1d) were putatively assigned to the flavonoid family, whose content is notoriously abundant in sweet cherries [9]. These results prove that HRMAS NMR spectroscopy allows us to identify and investigate the primary metabolome of Bracigliano PGI fresh cherries. Additionally, as discussed in depth in the following paragraph, we observed that the spectra of cherries belonging to the same variety exhibited a very similar profile, thus confirming the high reproducibility and reliability of the proposed method.

#### 3.1.2. Varietal Identification

Another important aspect is to ascertain the capacity to discriminate among the three investigated cherry varieties as a function of their NMR-based primary metabolome. Therefore, by only visually comparing representative proton spectra of SPE, PRI, and PAL berries, it emerged that the concentration of certain compounds varied according to the specific variety. This outcome was proved systematically in most of the replicates (in Figure 1 are shown 2/7 replicates per cherry variety).

In order to prove such an important finding through an objective approach, proton spectra were divided into buckets, integrated, and included in a first data matrix, which was then processed through various multivariate statistical analyses. Initially, the Principal Component Analysis (PCA) was applied. The latter produces two interrelated outputs, referred to as the score plot (related to the observations) and loading plot (related to the variables), and is very advantageous since it enables a rapid exploration of the internal variance among samples. PCA is an unsupervised test usually adopted in metabolomics since it allows a rapid identification of dissimilarities between different sample types, facilitating the identification of peculiar metabolic traits.

**Figure 2:**
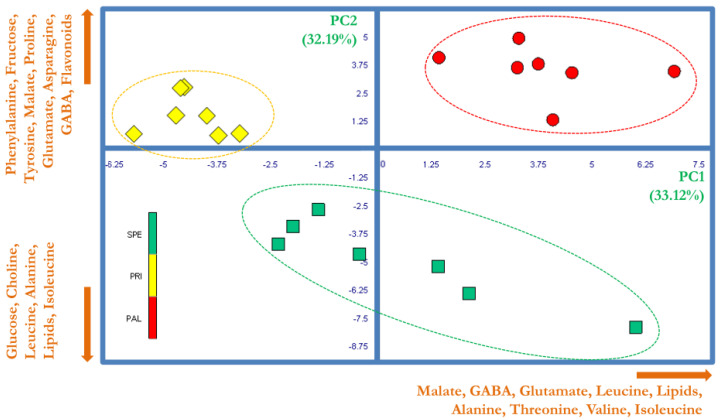
PCA score plot based on ^1^H HRMAS NMR spectra of Bracigliano PGI cherry varieties Spernocchia (SPE, green squares), Principe (PRI, yellow rhombuses), and Pallaccia (PAL, red circles). The ellipses have been arbitrarily generated to emphasize the groups’ separation. The names and orthogonal directions of the most significant loading vectors involved in the samples’ discrimination are reported in orange along the edges.

PCA was performed on a data matrix composed of 21 observations (7 replicates for SPE, PAL, and PRI) associated with 47 variables represented by the normalized integrations of spectral buckets. Figure 2 shows the score plot generated by combining the PC1 and PC2 (explaining 33.12% and 32.19% of the total variability, respectively). This combination confirmed that HRMAS spectra can easily discriminate the investigated Bracigliano sweet cherries. In fact, since all the replicates of SPE, PRI, and PAL were positioned in three distinct spatial regions in the area defined by the PC1 and PC2, the variability of specific NMR signals strongly correlated with the variety type. Moreover, the way the single replicates were collocated by PCA indicated that the inter-class variance was much larger than the intra-class one, thus suggesting the proposed analytical approach as a suitable tool to classify Bracigliano PGI sweet cherries. The exam of the PCA loading plot permitted the individuation of the variables mostly represented by the linear combinations PC1 and PC2. Then, such a selection was assessed by the ANOVA test to identify only those significantly discriminating the different cherry varieties.

In detail, it emerged a neat differentiation along the PC1 between PRI berries and the other two varieties, since Principe cherries were characterized by a significantly lower amount of malate, GABA, glutamate, leucine, lipids, alanine, threonine, valine, and isoleucine. Moreover, the PAL and SPE varieties were clearly differentiated along the PC2 axis. In fact, as compared to SPE, PAL exhibited a relatively higher content of phenylalanine, fructose, tyrosine, malate, proline, glutamate, asparagine, GABA, and flavonoids (variables correlating positively with the PC2), along with a relatively lower content of glucose, choline, leucine, alanine, lipids, and isoleucine (variables correlating negatively with the PC2).

HRMAS data of sweet cherry samples were also evaluated by a PLS-DA cross-validation test. Supplementary Figure S1a–c shows the most important outputs of one representative cross-validation test, including (i) a score plot with validation scores and groups’ centroids, (ii) a cross-validation table showing the extent of unknown samples classified correctly, and (iii) the ROC curve. PLS-DA further validated our conclusions, since 100% of “unknown” replicates were classified correctly in each of all the five cross-validation rounds. This helps to bolster the evidence that the botanical origin determines a specific metabolomic fingerprint in studied cherries, which can be recognized by HRMAS NMR.

Our results demonstrated that HRMAS NMR was capable of successfully classifying the studied Bracigliano PGI cherry varieties. This was particularly true since the investigated berries were produced under similar conditions (vintage, soil type, pedoclimatic factors, and harvesting procedures/timelines) and the botanical origin was the predominant factor. These findings are extremely promising and permit us to consider HRMAS as a valuable, reliable, and fully compatible technique for analyzing the primary metabolome of cherries and conducting a botanical traceability of Bracigliano PGI sweet cherries.

### 3.2. Analysis of Commercial, Chemical, and Nutraceutical Parameters

Chemical and quality parameters (pH, titratable acidity, soluble solids, dry weight, and fruit hardness) of the investigated Bracigliano GPI sweet cherry varieties were also assessed to further characterize these products (Table 1). The contents of soluble solids, mostly represented by sugars and expressed in °Bx, were very similar for all varieties, with an average value of 19 °Bx, with SPE showing the relatively lower average value. Since the Brix values for sweet cherries typically range between 13 and 23 °Bx, the analyzed Bracigliano PGI sweet cherries exhibited satisfying results, which were relatively higher than the average. This aspect is particularly relevant, as it correlates with the presence of certain oligosaccharides, contributing to sweetness and making more desirable several organoleptic properties.

While the fresh weight of sweet cherries is important from a commercial point of view, the dry weight is often related to its sensory characteristics. The average dry weights of PAL, PRI, and SPE were 24.07, 25.14, and 20.02%, respectively (Table 1). Since the Brix degree of a fruit reflects mostly the content of soluble sugars in its liquid phase, a certain correlation with dry weight was expected. Interestingly, SPE, which was the one with the lowest °Bx, exhibited the lowest dry weight.

The firmness of sweet cherries is another important sensorial aspect, being related to fruit crispness. As shown in Table 1, the variety producing the fruit with the highest firmness was PRI, with an average of 820 g/cm^2^, followed by SPE (761 g/cm^2^) and PAL (666 g/cm^2^). The measured firmness values fell within the typical range of 600 to 1100 g/cm^2^ [19].

The titratable acidity assay revealed that PAL was distinctly and significantly different from the other ones, with a value of 16.88 g/L, while PRI and SPE exhibited the values of 9.46 and 9.14 g/L, respectively (Table 1). Since malic acid accounts for 65–80% of the total organic acids in sweet cherries, it dictates their titratable acidity. Consistently, it is interesting to note that HRMAS NMR data (Figure 2) revealed the largest amount of malate in PAL. Studies evaluating the relationship between soluble solids content and total acidity in sweet cherries indicate a decrease in consumers’ preference when the ratio between these parameters exceeds a certain threshold [20].

The pH in sweet cherries typically ranges from 3.7 to 4.2 [21] and is expected to vary, depending on the specific variety. The pHs for examined cherries were included within 3.56 and 3.8, thus falling within an acidic range, with values slightly more acidic than the average for sweet cherries. Specifically, the variety with the lowest pH was PAL (3.34), followed by SPE (3.46) and PRI (3.49) (Table 1). These values are considered suitable for copigmentation in red cherries [22]. As expected, there was a good correlation between pH and titratable acidity. In fact, PAL was the variety with the highest titratable acidity and the lowest pH.

Sweet cherries are fruits notoriously rich in bioactive compounds with a nutraceutical action, such as phenols and polyphenols. In particular, phenolic compounds, which are concentrated in the berry skin, contribute to many sensory and organoleptic traits, and their accumulation is strongly related to the maturation process [23].

The variety with the highest content in total phenols (TPs) was PAL, with 1.201 mg GAE/g fw, followed by PRI and SPE, with 0.917 and 0.776 mg GAE/g fw, respectively (Table 2). The differences observed between the three varieties were undoubtedly attributable to the distinct genotypes. However, considering that the average contents of TP for sweet cherries range between 0.5831 and 1.1441 mg GAE/g fw [24,25,26,27,28], the values observed for all the studied cherry types resulted above the average, thus proving their relevant nutraceutical quality. Importantly, the pedoclimatic and environmental conditions persisting in the territory of Bracigliano may have played an important role in defining the phenolic composition of each of the species examined.

The free radical scavenging activity via DPPH was assessed for all the studied cherry types. In line with TP results, all investigated varieties were characterized by high levels of antioxidant agents, exceeding 1000 μg AAE/g fw. PAL predominated with values of 1145.33 μg AAE/g fw, followed by PRI with 1101.07 μg AAE/g fw, and finally by SPE with 1065.07 μg AAE/g fw (Table 2). Considering that the typical values of free radical scavenging activity of sweet cherries are rarely higher than 1000 μg AAE/g fw [13], it is important to emphasize that all the investigated cherry types exhibited values slightly exceeding the average. It is further noteworthy that the data obtained from the DPPH test were entirely consistent with those on TP, since also in terms of antioxidants, PAL exhibited the highest value. Such an outcome further corroborates the high nutraceutical quality of the investigated PGI cherries.

It is noteworthy that, although Folin–Ciocalteu and DPPH assays only provide a general evaluation of the contents of total phenols and antioxidants, they represent a valid, rapid, and reproducible approach to supply an overall quantification of these compound classes and evaluate the nutraceutical potential of the consumption of specific food products.

### 3.3. Clustered Heatmap of Combined Data

The heatmap technique, which is another “unsupervised” method, was applied to process all the data that contributed significantly to discriminating the investigated Bracigliano PGI sweet cherry types. The clustered heatmap is very advantageous because it clearly and directly highlights the semi-quantitative response (from blue to red) of the variables mostly involved in the differentiation between the sample types. Moreover, it reports a clusterization of both variables (dendrogram on the left-hand side of the figure) and samples (dendrogram on the top of the figure) based on their mutual similarity, providing two sets of dendrograms where objects are linked to one another depending on their progressively decreasing similarity. Consequently, in the case of sample clusterization, the more similar the values for the compared variables, the closer the observations are placed in this structure.

**Figure 3:**
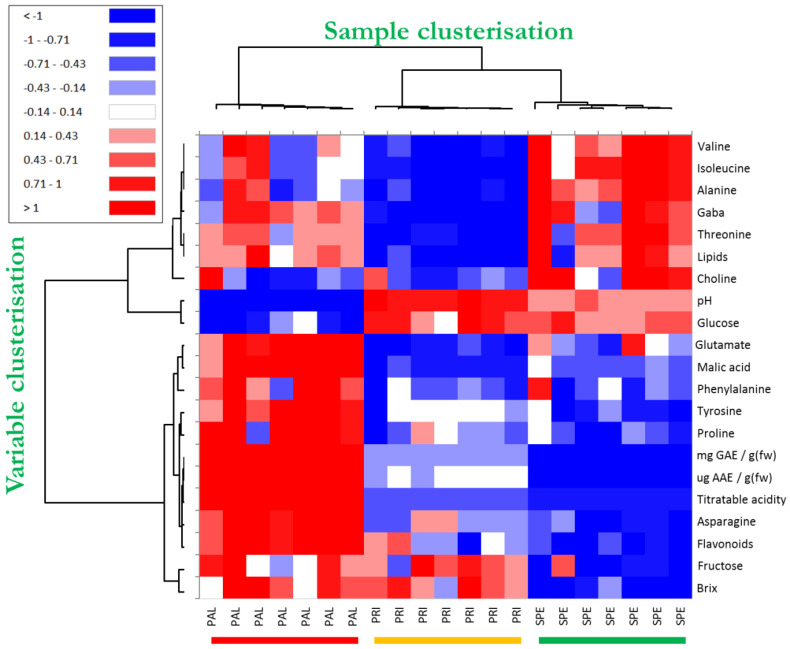
Clustered heatmap based on the variables significantly involved in the discrimination among the Bracigliano PGI cherry varieties Spernocchia (SPE, green), Principe (PRI, yellow), and Pallaccia (PAL, red) and including NMR, nutraceutical, and chemical data. The values were centered, scaled, and displayed according to a color gradient ranging from red (relatively high values) to blue (relatively low values), with white representing intermediate values.

The clustered heatmap of the studied sweet cherry samples is shown in Figure 3. By focusing on the sample dendrogram on the top of the figure, it is interesting to note that the first knot clearly separated all the PAL samples from those of the other two cherry varieties by generating two distinct macroclusters. Then, the second knot separated SPE from PRI by developing two further subclusters. Interestingly, the color modulation permitted us to visually appreciate the neat differences within the studied cherries for all the sets of results. The fact that all the replicates belonging to the same cherry variety were grouped in the same cluster proved that the adopted method was reliable to discriminate the cherry varieties. The discriminatory power of the matrix composed of all the variables was corroborated by the PLS-DA cross-validation test (Supplementary Figure S1d–f), in which 100% of “unknown” replicates were again classified correctly in each of all the five cross-validation rounds. Such a finding was further evidence that all the experimental data considered in this study (nutraceuticals, chemical quality, and HRMAS NMR) provided peculiar traits of the investigated sweet cherries by permitting their identification. Finally, the exam of the tree structure on the left put in evidence the correlations existing among all the variables. In particular, it emerged in PAL a direct correlation among flavonoids, TP, and AAE, as well as between fructose and °Bx. Likewise, the heatmap also highlighted the significant role of amino acids, such as valine, isoleucine, alanine, GABA, and threonine, in discriminating PRI from SPE. In Supporting Table S3, the Pearson correlation matrix of variables mostly involved in the discrimination among investigated sweet cherry types is shown. In line with the heatmap results, in red and in blue are highlighted the most intense positive and negative correlations.

## 4. Conclusions

A large interest is related to the quality of sweet cherries due to their large use for fresh consumption and industrial purposes, for their pleasant sensory properties, and for the health effects related to their consumption. In the Italian region of Campania, the sweet cherries of Bracigliano are very precious and promising, as proved by the fact that in 2023, they were assigned the important European label PGI. On this basis, we characterized sweet cherry types produced by Pallaccia, Spernocchia, and Principe plants, which are the most important and representative Bracigliano varieties included in the PGI label. Innovatively, and for the first time to the best of our knowledge, we used HRMAS NMR spectroscopy, which is a very powerful but still deeply unexplored NMR technique, to investigate the primary metabolome of the selected PGI cherry berries. Surprisingly, we demonstrated that HRMAS NMR is suitable to analyze fresh sweet cherries and can provide very useful data. In detail, we unveiled the NMR-based primary metabolome of fresh Bracigliano PGI species, identifying the most important amino acids, carbohydrates, alcohols, and organic acids as well as individuating peaks, which we assigned to the flavonoid family. Moreover, the use of chemometrics (ANOVA, PCA, and PLS-DA elaborations) highlighted diagnostic and significant differences among the berries produced by Pallaccia, Spernocchia, and Principe varieties, thus permitting the definition of a variety-specific compositional fingerprint. We also characterized the products in terms of chemical and nutraceutical quality, detecting a relatively elevated content in healthy compounds, such as total phenols and antioxidant agents, and defining Pallaccia cherries as the healthiest ones. The HEATMAP clusterization permitted finding interesting correlations between HRMAS-NMR data and quality parameters.

Concluding, our results pave the way to the creation of a more dense and reliable spectral database of Bracigliano sweet cherries, useful to conduct traceability studies, protect consumers from fraud, and help producers to promote and certify the quality of their products. Such an outcome is very important because the Bracigliano PGI sweet cherries have been awarded the prestigious PGI label only in 2023. In the future, more robustness will be provided to the method and to the spectral database by including a larger number of replicates for HRMAS metabolomics. Moreover, this analytical approach will be extended to PGI Bracigliano cherries collected from both different vintages and different soils in order to verify the reproducibility of the outcomes and find possible correlations with the terroir.

## Figures and Tables

**Table 1:** Chemical and quality parameters (pH, titratable acidity, soluble solids, and dry weight) of the investigated Bracigliano GPI cherry varieties Spernocchia, Principe, and Pallaccia (averages ± standard deviations with bold letters indicating the outcome of the ANOVA test at a *p*-value < 0.05).

	pH	Titratable Acidity (g/L)	Soluble Sugars (°Bx)	Dry Weight (%)	Firmness (g/cm^2^)
**Pallaccia**	3.35 ± 0.01 **c**	16.88 ± 0.06 **a**	19.78 ± 0.47 **a**	24.07 ± 0.11 **b**	666 ± 73 **b**
**Principe**	3.51 ± 0.01 **a**	9.46 ± 0.07 **b**	19.65 ± 0.30 **a**	25.14 ± 0.56 **a**	820 ± 83 **a**
**Spernocchia**	3.47 ± 0.01 **b**	9.14 ± 0.03 **c**	18.65 ± 0.39 **b**	20.02 ± 0.04 **c**	761 ± 88 **ab**

**Table 2:** Contents of total phenols (mg GAE/g fresh weight) and antioxidants (µg AAE/g fresh weight) in the Bracigliano PGI cherry varieties Spernocchia, Principe, and Pallaccia (averages ± standard deviations with bold letters indicating the outcome of the ANOVA test at a *p*-value < 0.05).

	Total Phenols (mg GAE/g)	Free Radical Scavenging Activity via DPPH (µg AAE/g)
**Pallaccia**	1.210 ± 0.006 **a**	1152.9 ± 2.9 **a**
**Principe**	0.924 ± 0.004 **b**	1110.6 ± 4.2 **b**
**Spernocchia**	0.782 ± 0.004 **c**	1075.8 ± 2.8 **c**

## Data Availability

The original contributions presented in the study are included in the article/Supplementary Materials, further inquiries can be directed to the corresponding author.

## Supplementary Materials

The following supporting information can be downloaded at: https://www.mdpi.com/article/10.3390/foods14122120/s1, Table S1. Principal characteristics of the PGI Bracigliano cherry berries Spernocchia, Pallaccia, and Principe; Table S2. Assignment of the proton and the corresponding carbon peaks detected by 1D and 2D HRMAS NMR spectra and representing the primary metabolome of sweet cherry berries; Table S3. Correlation matrix investigating the variables mostly involved in the discrimination among the Bracigliano PGI cherry varieties Spernocchia, Principe, and Pallaccia and including NMR, nutraceutical, and chemical data. The most intense positive and negative correlations are highlighted in red and in blue, respectively; Figure S1. Representative PLS-DA cross-validations, based on NMR spectra (set on top) and all the data (set on the bottom), to discriminate the Bracigliano PGI cherry varieties Spernocchia (SPE, green), Principe (PRI, yellow), and Pallaccia (PAL, red). The figure includes cross-validation score plots (a,d), the receiver operator characteristics curve (ROCs; b,e), and the cross-validation results (c,f).
