# Supplementary material for: HRMAS NMR Spectroscopy to Identify the Primary Metabolome of Bracigliano PGI Sweet Cherries and Correlate It with Nutraceutical and Quality Parameters
Source: Foods. 2025 Jun 17;14(12):2120. doi: 10.3390/foods14122120 (PMC12191911; doi:10.3390/foods14122120)
Supplement: Supplementary file 1 [file foods-14-02120-s001.zip › foods-3647549-supplementary.pdf]

Supporting information for the

Original research paper

"HRMAS NMR spectroscopy to identify the primary metabolome of Bracigliano PGI sweet cherries and correlate it with nutraceutical and quality parameters"

Domenico Liguori & Pierluigi Mazzei*

Department of Pharmacy, University of Salerno, 84084 Fisciano, Italy

*Correspondence: Pierluigi Mazzei, Orcid 0000-0002-5312-4969, pmazzei@unisa.it

Tables: 3

Figure: 1

Supporting Table S1. Principal characteristics of the PGI Bracigliano cherry berries Spernocchia, Pallaccia and Principe

	SPERNOCCHIA (SPE)	PALLACCIA (PAL)	PRINCIPE (PRI)
**Geographical origin**	Bracigliano (Salerno, Italy)	Bracigliano (Salerno, Italy)	Bracigliano (Salerno, Italy)
**Size**	high	high	moderately high
**Shape**	Heart-shaped	Elongated Spherical	Spherical
**Colour**	Dark ruby red	Red tending towards black	From light red to dark red
**Mesocarp**	Hard and crunchy	Hard and crunchy	Hard and crunchy
**Taste**	Sweet	Medium Sour	Sweet
**Shelf-life**	high	Moderate	Moderately high
**Harvest Period**	Early June	End of May	Mid-may
**Peduncle**	Long	Short	long

Supporting Table S2. Assignment of the proton and the corresponding carbon peaks detected by 1D and 2D HRMAS NMR spectra and representing the primary metabolome of sweet cherry berries

Compound name	^{1}H Chemical Shift (*ppm*)	^{13}C Chemical Shift (*ppm*)
**Amino acids**		
alanine	1.47	16.4
asparagine	2.909	37.27
GABA	1.926/ 2.991/ 3.095	26.6/ 37.2 / 42.1
glutamate	1.957/ 2.242	29/ 35.5
glutamine	2.141/ 2.475	26.56/ 31.21
isoleucine	0.923	13.9
leucine	1.751	42.5
phenylalanine	7.316/ 7.417	132.03/ 131.76
proline	2.031/ 2.19/ 3.312	26.3/ 31.5/ 48.7
threonine	1.33/ 4.32	22.1/ 68.1
tryptophan	7.19/ 7.278/ 7.3124	122.24/ 124.87/ 127.82
tyrosine	6.42	118.28
valine	0.98/ 1.039	19.3/ 20.8
**Carbohydrates**		
fructose	4.114/ 4.016/ 3.986/ 3.565	77.8/ 66.1/ 71.9/ 65.3/
glucose	5.231 (α) / 4.63 (β) / 3.51/ 3.236	94.6 (α)/ 98.4 (β)/ 74.1/ 76.5
sucrose	5.413/ 4.207	95.2/ 79.1
**Organic acids**		
hydroxycinnamic acid	7.032	126.6
isocitrate	2.401	49.5
malate	2.626/ 2.817/ 4.42	42.94/ 42.94/ 70.67
**Alcohols**		
ethanol	1.16	17.2
methanol	3.34	48.1
**Other compounds**		
choline	3.18	46.45
ethylacetate	1.203	13.8
flavonoids *	6.08/ 6.17/ 6.91/ 7.65	112/ 117/ 132/ 128
lipids	0.9/ 1.3/ 1.6	14.2/ 29.1/ 34.3

* *The signals resonating at these frequencies were putatively and generically attributed to the family of flavonoids.*

Supporting Table S3. Correlation matrix investigating the variables mostly involved in the discrimination among the Bracigliano PGI cherry varieties Spernocchia, Principe and Pallaccia and including NMR, nutraceutical and chemical data. The most intense positive and negative correlations are highlighted in red and in blue, respectively.

Malic acid	1																					
Gaba	0.485	1.000																				
Glutammate	0.898	0.691	1.000																			
Leucine	0.022	0.836	0.248	1.000																		
Alanine	0.129	0.874	0.331	0.942	1.000																	
Threonine	0.376	0.848	0.570	0.754	0.883	1.000																
Lipids	0.521	0.874	0.641	0.730	0.837	0.960	1.000															
Valine	0.300	0.903	0.449	0.891	0.962	0.898	0.893	1.000														
Isoleucine	0.191	0.850	0.353	0.883	0.971	0.925	0.890	0.972	1.000													
Phenylalanine	0.689	0.382	0.675	0.182	0.238	0.415	0.458	0.369	0.243	1.000												
Flavonoids	0.768	0.034	0.584	-0.383	-0.295	-0.084	0.057	-0.080	-0.243	0.658	1.000											
Tyrosine	0.748	0.089	0.667	-0.247	-0.217	0.085	0.195	-0.032	-0.141	0.777	0.833	1.000										
Fructose	0.157	-0.396	0.153	-0.546	-0.585	-0.523	-0.533	-0.563	-0.656	0.232	0.467	0.403	1.000									
Glucose	-0.596	-0.197	-0.638	0.150	0.005	-0.290	-0.279	-0.135	-0.070	-0.574	-0.598	-0.581	-0.441	1.000								
Choline	-0.274	0.572	0.000	0.724	0.673	0.465	0.372	0.599	0.593	-0.186	-0.506	-0.591	-0.406	0.094	1.000							
Asparagine	0.793	0.089	0.709	-0.354	-0.310	-0.061	0.063	-0.177	-0.310	0.633	0.848	0.782	0.591	-0.615	-0.426	1.000						
Proline	0.665	0.216	0.754	-0.174	-0.177	0.092	0.144	-0.050	-0.163	0.628	0.674	0.790	0.522	-0.723	-0.202	0.821	1.000					
pH	-0.940	-0.488	-0.913	-0.004	-0.142	-0.422	-0.509	-0.309	-0.214	-0.648	-0.728	-0.694	-0.226	0.804	0.146	-0.762	-0.746	1.000				
Titr. acidity	0.912	0.265	0.834	-0.216	-0.111	0.191	0.302	0.080	-0.039	0.655	0.854	0.803	0.428	-0.817	-0.328	0.870	0.805	-0.954	1.000			
mg GAE/g	0.812	0.031	0.689	-0.418	-0.353	-0.057	0.075	-0.158	-0.288	0.593	0.911	0.847	0.577	-0.750	-0.495	0.912	0.807	-0.830	0.957	1.000		
µg AAE/g	0.748	-0.085	0.601	-0.499	-0.463	-0.173	-0.029	-0.275	-0.402	0.543	0.900	0.846	0.611	-0.680	-0.572	0.900	0.783	-0.746	0.906	0.988	1.000	
°Bx	0.377	-0.423	0.152	-0.591	-0.599	-0.447	-0.318	-0.460	-0.554	0.300	0.688	0.651	0.669	-0.318	-0.771	0.549	0.341	-0.297	0.534	0.709	0.772	1
	Malic acid	Gaba	Glutammate	Leucine	Alanine	Threonine	Lipids	Valine	Isoleucine	Phenylalanine	Flavonoids	Tyrosine	Fructose	Glucose	Choline	Asparagine	Proline	pH	Titr. acidity	mg GAE/g	µg AAE/g	°Bx

HRMAS NMR spectra

(a)

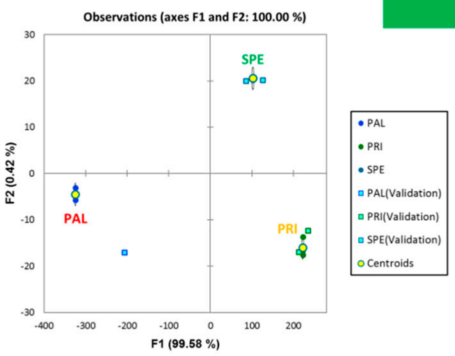

(b)

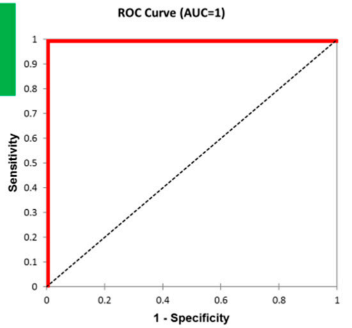

(c)

from \ to	PAL	PRI	SPE	Total	% correct
PAL	2	0	0	2	100.00%
PRI	0	2	0	2	100.00%
SPE	0	0	2	2	100.00%
Total	2	2	2	6	100.00%

All data

(d)

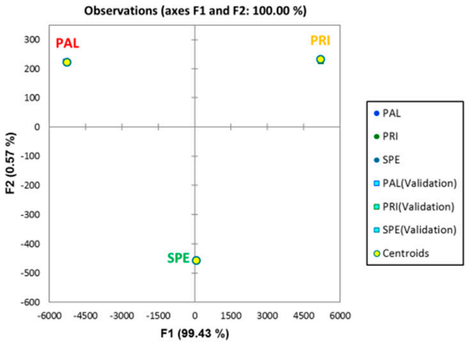

(e)

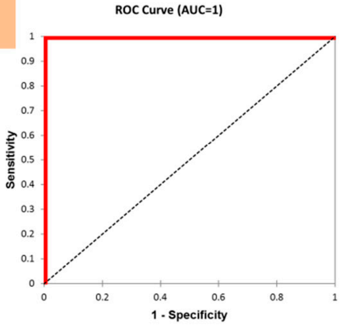

(f)

from \ to	PAL	PRI	SPE	Total	% correct
PAL	2	0	0	2	100.00%
PRI	0	2	0	2	100.00%
SPE	0	0	2	2	100.00%
Total	2	2	2	6	100.00%

Supporting Figure S1. Representative PLS-DA cross-validations, based on NMR spectra (set on top) and all the data (set on the bottom), to discriminate the Bracigliano PGI cherry varieties Spernocchia (SPE, green), Principe (PRI, yellow) and Pallaccia (PAL, red). The figure includes cross-validation score-plots (a and d), the receiver operator characteristics curve (ROCs; b and e) and the cross-validation results (c and f).
